# Lurasidone in treatment of manic episode

**DOI:** 10.1192/j.eurpsy.2022.1013

**Published:** 2022-09-01

**Authors:** I. Sánchez-Rivero

**Affiliations:** Hospital Doctor Rodríguez Lafora, Unidad De Cuidados Psiquiátricos Prolongados, Madrid, Spain

**Keywords:** manic episode, Treatment, lurasidone, bipolar disorder

## Abstract

**Introduction:**

Lurasidone is an atypical antipsychotic used in the treatment of schizophrenia and bipolar depression. Both indications are approved by the FDA nowadays, whereas in Europe it is only approved for schizophrenia. Lurasidone has been barely studied for the treatment of acute mania, nonetheless it is sometimes used off-label.

**Objectives:**

A case of a patient with a manic episode treated with lurasidone is presented, in order to provide further evidence on this topic.

**Methods:**

The patient is a 43 year-old-woman with diagnosis of type I bipolar disorder, personality disorder and borderline intellectual functioning, resident in our Hospital’s long-stay psychiatric rehabilitation unit. She was previously under treatment with venlafaxine 75 mg/day, valproate 1500 mg/day and levomepromazine 25 mg on demand; remaining stable for months. The patient presented an episode consisting on agitation, irritability, verbiage, tachyphase, verbal aggressiveness and behavioral disturbances. Psysical restraint was needed for one day long and zuclopenthixol acetate 50 mg IM was administered twice within 5 days for the acute agitation. Venlafaxine was immediately withdrawn and lurasidone was progressively introduced up to 111 mg daily.

**Results:**

Approximately 3 weeks after the treatment adjustment, the patient reached the psychopatological stabilty.

**Conclusions:**

Antidepressive withdrawal and introduction of Lurasidone were effective to treat the acute manic episode in this patient. It has been previosuly suggested that lurasidone caused improvement in emergent manic symptoms in patients with bipolar depression, and in subsyndromal hypomanic symptoms in patients with mixed features of depression. However, no studies have been made yet to evaluate the efficacy of lurasidone in acute mania.

**Disclosure:**

I received financing from Angelini Pharma, Casen Recordati, Janssen, Exeltis and Otsuka.

